# Cognitive Impairment Profiles in Dementia Stages: The ACAD Pilot Study Focusing on Korean Americans

**DOI:** 10.1002/alz70858_106748

**Published:** 2025-12-26

**Authors:** Haeok Lee, Eun Hyun Seo, Hyun‐Sik Yang, Yeunjoo E. Song, Nayoung Kim, Hoowon Kim, Younhee Kang, Kyungmin Kim, Yong Jeong, Gyungah R Jun, Yun‐Beom Choi, Kun Ho Lee, The Asian Cohort for Alzheimer's Disease Study

**Affiliations:** ^1^ Meyers College of Nursing, New York University, New York, NY, USA; ^2^ Gwangju Alzheimer's and Related Dementia (GARD) Cohort Research Center, Chosun University, Gwangju, Korea, Republic of (South); ^3^ Premedical Science, College of Medicine, Chosun University, Gwangju, Korea, Republic of (South); ^4^ Department of Neurology, Harvard Medical School, Boston, MA, USA; ^5^ Brigham and Women's Hospital, Boston, MA, USA; ^6^ Department of Population and Quantitative Health Sciences, Institute for Computational Biology, Case Western Reserve University, Cleveland, OH, USA; ^7^ Meyers College of Nursing, New York University, NY, NY, USA; ^8^ Department of Neurology, School of Medicine, Chosun University/Chosun University Hospital, Gwangju, Korea, Republic of (South); ^9^ National Research Center for Dementia, Chosun University, Gwangju, Korea, Republic of (South); ^10^ College of Nursing, Ewha Womans University, Seoul, Korea, Republic of (South); ^11^ Department of Child Development and Family Studies, Seoul, Seoul, Korea, Republic of (South); ^12^ Graduate School of Medical Science & Engineering, KAIST, Daejeon, Korea, Republic of (South); ^13^ Department of Bio and Engineering, KAIST, Daejeon, Korea, Republic of (South); ^14^ Department of Biostatistics, Boston University School of Public Health, Boston, MA, USA; ^15^ Department of Medicine (Biomedical Genetics), Boston University Chobanian & Avedisian School of Medicine, Boston, MA, USA; ^16^ Department of Neurology, Rutgers New Jersey Medical School, Newark, NJ & Englewood Health, Englewood, NJ, USA; ^17^ Englewood Health, Englewood, NJ, USA; ^18^ Biomedical Science, Chosun University, Gwangju, Gwangju, Korea, Republic of (South)

## Abstract

**Background:**

Alzheimer's disease and related dementia (ADRD) is rapidly increasing among the Asian American population. Studies have shown that risk factors for ADRD are not uniform across the population, and vary significantly depending on factors including ethnicity, socio‐environment, and individual clinical presentation. More research is needed to address the unique challenges faced by Asian Americans, including identifying specific risk factors within this diverse population. The purpose of this paper is to provide group‐specific risk factors for ADRD among Korean American older adult groups.

**Methods:**

A pilot cohort study of 54 Korean American adults ≥60 years old completed cognitive, socio‐cultural, functional, and neurological assessments to make a diagnosis of normal control (NC), subjective cognitive complaints (SCC), mild cognitive impairment (MCI), or dementia (DEM). Selected risk factors were compared between groups (NC‐SCC vs. MCI‐DEM) by either a t‐test or a Chi‐squared test.

**Results:**

As a part of the Asian Cohort for Alzheimer's Disease pilot study, 54 participants, 67% were females, the mean age of all the participants was 70.1 ( ± 7.2) years, ranging from 60 to 101, and 17% had completed elementary school education. Thirty‐three (33) were diagnosed as NC‐SCC while 21 were diagnosed with MCI‐DEM. Compared with the NC‐SCC group, the MCI‐DEM group showed a significant decline in cognitive performance (*p* < .001) measured by the modified mini‐mental status examination, common objective test, fluency test, and clock drawing test. The NC‐SCC group had better functional activities of daily living (*p* < .001) compared to the MCI‐DEM group. The MCI‐DEM group was older (*p* = .003) and had higher depression levels than the NC‐SCC group (*p* = .001). However, no significant difference was observed with other factors for ADRD including gender, education, length of stay in the U.S., English proficiency, hypertension, diabetes, and high cholesterol.

**Conclusions:**

The study demonstrates that the MIC‐DEM group experienced a significant cognitive decline compared to the NC‐SCC group and that age and depression, are significantly elevated in the MCI‐DEM group compared to the NC‐SCC group. Future research should replicate and expand upon our findings to enhance understanding of group specific‐risk factors and cognitive profiles in dementia staging to improve the early detection of ADRD.

